# Measuring and Validating the Levels of Brain-Derived Neurotrophic Factor in Human Serum

**DOI:** 10.1523/ENEURO.0419-17.2018

**Published:** 2018-03-22

**Authors:** Yvonne Naegelin, Hayley Dingsdale, Katharina Säuberli, Sabine Schädelin, Ludwig Kappos, Yves-Alain Barde

**Affiliations:** 1 School of Biosciences, Cardiff University, Cardiff CF10 3AX, United Kingdom; 2 Department of Neurology, University Hospital Basel, Basel 4031, Switzerland; 3 Clinical Trial Unit, Department of Clinical Research, University Hospital Basel, Basel 4031, Switzerland

**Keywords:** antibodies, BDNF, biomarkers, ELISA, platelets, Western blotting

## Abstract

Brain-derived neurotrophic factor (BDNF) secreted by neurons is a significant component of synaptic plasticity. In humans, it is also present in blood platelets where it accumulates following its biosynthesis in megakaryocytes. BDNF levels are thus readily detectable in human serum and it has been abundantly speculated that they may somehow serve as an indicator of brain function. However, there is a great deal of uncertainty with regard to the range of BDNF levels that can be considered normal, how stable these values are over time and even whether BDNF levels can be reliably measured in serum. Using monoclonal antibodies and a sandwich ELISA, this study reports on BDNF levels in the serum of 259 volunteers with a mean value of 32.69 ± 8.33 ng/ml (SD). The mean value for the same cohort after 12 months was not significantly different (*N* = 226, 32.97 ± 8.36 ng/ml SD, *p* = 0.19). Power analysis of these values indicates that relatively large cohorts are necessary to identify significant differences, requiring a group size of 60 to detect a 20% change. The levels determined by ELISA could be validated by Western blot analyses using a BDNF monoclonal antibody. While no association was observed with gender, a weak, positive correlation was found with age. The overall conclusions are that BDNF levels can be reliably measured in human serum, that these levels are quite stable over one year, and that comparisons between two populations may only be meaningful if cohorts of sufficient sizes are assembled.

## Visual Abstract

**Figure:**
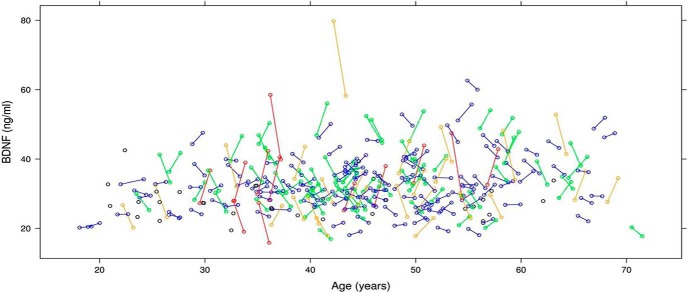

## Significance Statement

The presence of brain-derived neurotrophic factor (BDNF) in human blood has generated considerable interest as illustrated by the very large number of publications associating BDNF levels with various conditions affecting brain function, including depression and neurodegeneration. Yet a range of technical issues, together with a lack of plausible mechanisms explaining this association, raise questions as to the meaning and value of such measurements. This contribution deals with the feasibility of reliably measuring BDNF levels in human serum and gives indications of the size of cohorts to be recruited for meaningful differences to be observed between populations of interest.

## Introduction

Brain-derived neurotrophic factor (BDNF) is a member of the neurotrophin family involved in many aspects of neuronal function, as evidenced by a large number of animal experiments as well as human genetics (Zagrebelsky and Korte, 2014). BDNF is not only found in the brain, but also in circulating platelets in humans (Yamamoto and Gurney, 1990) and megakaryocytes have recently been reported to express the same transcripts as neurons (Chacón-Fernández et al., 2016). As BDNF is released from platelets during the process of blood coagulation, its levels can be readily measured in serum. Even if it is still entirely unclear why BDNF levels in platelets or serum should reflect brain levels (see Discussion), these levels have been associated with a number of conditions including depression (Karege et al., 2002; Sen et al., 2008), Huntington’s disease (Ciammola et al., 2007; Zuccato et al., 2011), and Alzheimer’s disease (Laske et al., 2006). However, whether or not BDNF levels can be reliably determined in human serum is increasingly a matter of debate (Zuccato et al., 2011). In addition, the very large range of reported individual values raises questions as to the plausibility of any disease conditions leading to significant differences in the levels of BDNF in serum. Concerns have also been expressed following reports that different commercially available ELISA kits for measuring BDNF in human serum give different values when the same samples of human serum are tested (Polacchini et al., 2015). There is then a pressing need to better understand if it is indeed possible to reliably measure BDNF concentrations in human serum before further considering the use of BDNF as a biomarker possibly reflecting disease conditions or for monitoring therapies.

The goal of this study was to address this question with an ELISA based on publically available monoclonal antibodies, using a cohort of 259 healthy volunteers retested after 12 months and to validate the ELISA results using independent Western blot analyses.

## Materials and Methods

### Serum samples

Serum samples from a cohort of volunteers were collected at the University Hospital of Basel (Switzerland) following a protocol approved by the local ethics committee and in accordance with the terms of the Declaration of Helsinki. All subjects (*N* = 259, 178 females, 81 males) gave written informed consent. All participants gave a first blood sample (defined as Initial), with most participants (226) agreeing to have a second sample drawn at a 12-month follow-up visit (defined as 12-month). The age range was between 18 and 70 years and the volunteers were of Northern Europe ethnicity. Blood was drawn from a cubital vein into a S-Monovette 7.5 ml Z tube (with clotting activator; Sarstedt), left to coagulate for 30 min at room temperature (RT) and centrifuged at 2000 × *g* at RT for 10 min. The serum supernatant was stored at -80°C in 0.5 ml aliquots after centrifugation within 1 h of blood sampling. Blood samples for platelet counts and hematocrit values were collected at the same time as blood samples used for the determination of BDNF levels. Time of blood sampling was between 8 A.M. and 12 P.M. in over 75% of the individuals examined.

### ELISA

#### Antibodies

ELISA measurements were performed using a combination of BDNF monoclonal antibodies designated mAb BDNF-#1 and mAb BDNF-#9 (Kolbeck et al., 1999). Both are available at the Developmental Studies Hybridoma Bank (DSHB), University of Iowa (http://dshb.biology.uiowa.edu). mAb-#1 was conjugated with biotin using sulfo-NHS-LC-Biotin (ThermoFisher Scientific, catalog number 21435). MAb-#9 was conjugated with horseradish peroxidase (HRP) using a peroxidase labeling kit (Roche, 11829696001) following manufacturer’s instructions or else provided conjugated by Icosagen.

#### Protocol

Pierce NeutrAvidin-coated high-capacity plates (ThermoFisher Scientific; 15509) were incubated for two hours at RT with 200 µl of 14 µg/ml biotin-conjugated mAb-#1 diluted in phosphate buffer (0.1% Triton X-100 in 0.1 M phosphate buffer: 0.1 M KH_2_PO_4_ and 0.1 M Na_2_HPO_4_; pH 7.6). Plates were then washed three times with blocking buffer [1% bovine serum albumin (BSA); Sigma A2153 in phosphate buffer], followed by the addition of 150 µl phosphate buffer. A total of 50 µl of either standards or diluted samples (both in blocking buffer) was then added to the plate followed by incubation for 3 h at RT on a rotary shaker. The standard was established using recombinant BDNF (Regeneron/Amgen) diluted in blocking buffer. Serum samples were tested at 1:20 dilution, but identical final values were also obtained with 1:10 and 1:40 dilutions. After 3 washes with phosphate buffer, 200 µl 1.25 μg/ml HRP-conjugated mAb-#9 diluted in blocking buffer was added and incubated for 3 h on a rotary shaker. The plate was then washed three times with phosphate buffer before the addition of 100 µl chemiluminescent substrate following manufacturer’s instructions (Chemiluminescence ELISA Substrate, Roche 11582950001), and the plate immediately analyzed with a microplate reader (FLUOstar OMEGA, BMG Labtech). Recovery experiments indicated 108.6% recovery of known amounts (30 ng/ml) of recombinant BDNF added to serum samples tested at the typical 1:20 dilution.

### Western blotting

One microliter human serum was applied onto a 4–12% NuPage Bis-Tris gradient gel (Invitrogen), alongside standards consisting of three different quantities of recombinant BDNF (Regeneron/Amgen), typically 15, 30, and 50 pg; 0.1% BSA was added to recombinant BDNF to approximate the composition of the serum samples and to improve the signal consistency between blots. The high level of serum albumin precluded the use of higher volumes of serum and albumin could not be selectively removed from the serum samples without also removing significant (>50%) amounts of serum BDNF. Proteins were transferred to 0.2 μm Protran nitrocellulose membranes (wet or semi-dry transfer at 80 V for 1 h at 4°C or 17V for 1.5 h at RT, respectively) and subsequently blocked for 1 h with a solution containing 3% BSA (Sigma A7906), 3% GE Healthcare ECL Prime Blocking reagent (GE Healthcare) in TBS-T (blocking solution). The membrane was then incubated overnight with primary antibody [1:2000 anti-BDNF (3C11, Icosagen)] in blocking solution. Following three 20-min washes with TBS-T, the membranes were incubated for 1 h with 1:2000 secondary antibody (HRP-conjugated goat anti-mouse IgG1; Invitrogen) in blocking solution, washed once with TBS-T, three times with Lumiglo Reserve wash solution (Insight Biotechnology) and developed using Lumiglo Reserve Chemiluminescent Substrate. Signals were recorded on a ChemiDoc MP Imaging System (Bio-Rad) and quantified using Image Lab V5.0 (Bio-Rad) and BDNF readings only considered when the *r*^2^ value of the standards was above 0.98, and the serum samples readings within the range of the standards. The limit of reliable BDNF detection by Western blotting was 15 ng/ml.

### Statistical analysis

As BDNF measurements were not normally distributed the values were log-transformed before analysis of significance. The association between initial BDNF values and both age and gender was assessed using a multivariable linear regression model. Hematocrit and platelet count were included as co-variables. Initial and 12-month BDNF measurements were modeled using generalised estimating equations to examine the association of BDNF to the various predictors while taking into account the dependence of data from the same volunteer. The *p* value between the first and second measurements was determined using paired *t* test.

## Results

**Figure 1.**
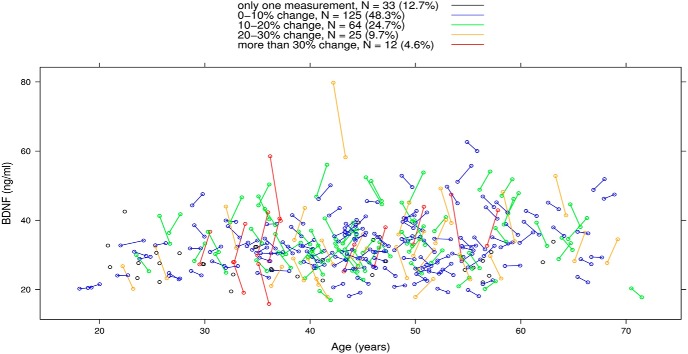
BDNF serum levels in a healthy cohort at Initial (*n* = 259) and 12-month (*n* = 226) visit. Measurements of the same subject are connected by a colored line to indicate percentage change in BDNF values between visits. Samples are separated on the *x*-axis by age in years.

**Figure 2.**
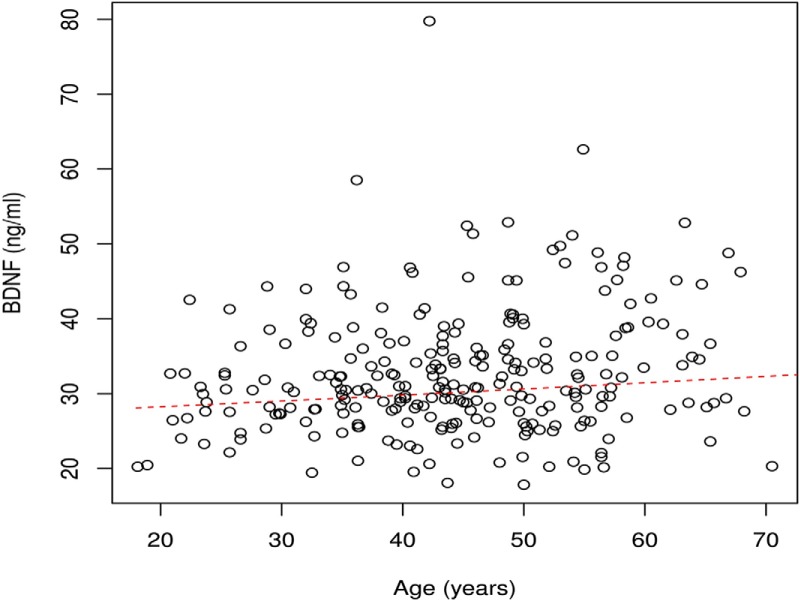
BDNF serum levels and age of participant. The red broken line indicates the estimated serum BDNF for a given age in an average volunteer.

**Figure 3.**
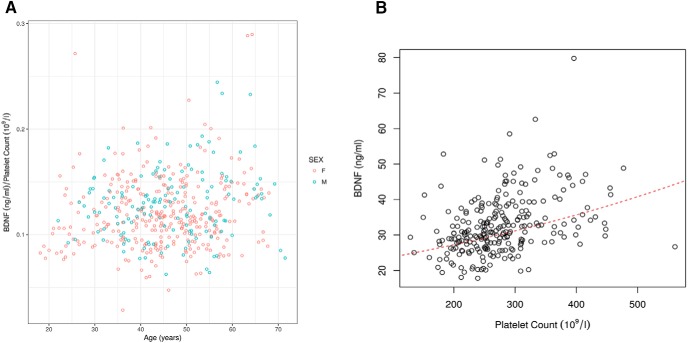
Correlation between BDNF values and platelet counts. ***A***, BDNF values per platelet plotted against age. ***B***, The red broken line indicates the estimated BDNF for a given platelet count in an average volunteer.

**Figure 4.**
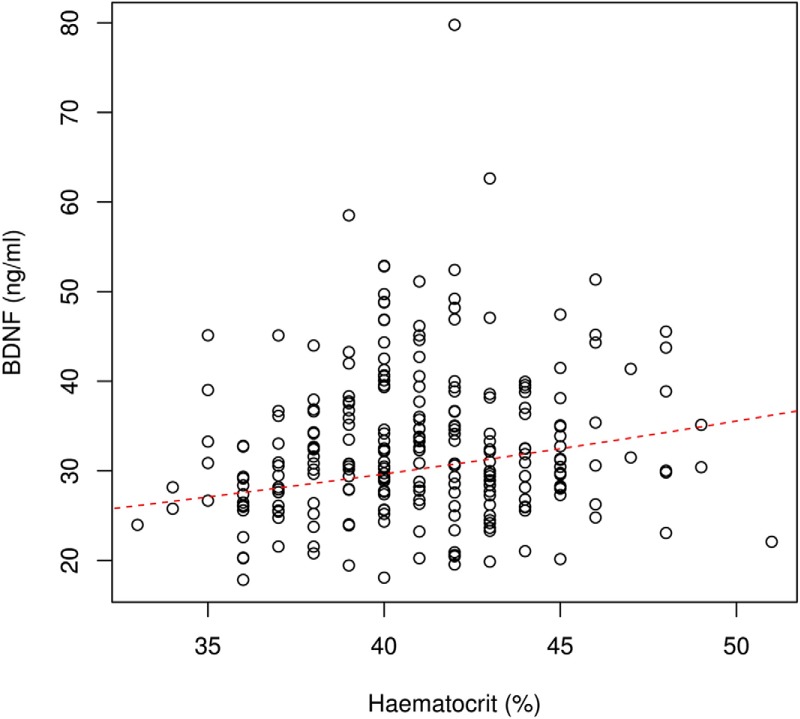
Correlation between BDNF values and hematocrit. The red broken line indicates the estimated BDNF for a given hematocrit in an average volunteer.

Given various reports on the heterogeneity of values obtained using commercially available BDNF ELISA kits (Polacchini et al., 2015), a slightly modified version of a previously described ELISA protocol was used (Kolbeck et al., 1999) based on publically available BDNF monoclonal antibodies (http://dshb.biology.uiowa.edu/). This assay has an interassay coefficient of variability of 8.8% as calculated with a single serum sample measured on 18 different plates [24.11 ± 2.12 ng/ml (SD); range, 20.63–28.66 ng/ml]. The intra-assay coefficient of variability was calculated as 9.7% based on 253 samples tested in triplicate. This ELISA was used to determine the serum BDNF levels in a cohort of volunteers (*n* = 259) and to monitor these levels in most of these volunteers (*n* = 226) over a 12-month period. Cohort demographics are listed in Table 1. Serum samples were collected following a strict protocol (see Methods), as the procedures used to collect the blood samples and to generate serum following blood collection are expected and indeed known to cause variability given that the bulk of BDNF eventually measured in serum is released from platelets (see Discussion). The values determined in the serum of 259 healthy volunteers are summarized in Table 2. The mean serum value (±SD) of BDNF at the first visit was 32.69 ± 8.33 ng/ml; the median value was 30.86 ng/ml. The mean serum value in participants 12 months later was 32.97 ± 8.36 ng/ml with a median of 32.27 ng/ml (Fig. 1), i.e., statistically indistinguishable from the mean levels determined from the initial blood samples (*p* = 0.19). When analyzed in detail, over half of the individuals retested after 12 months (55%) had BDNF serum values within 10% of their original reading (connected by blue lines seen in Fig. 1) with 37 individuals showing >20% changes. In Figure 1, they are connected by either yellow (20–30% change) or red lines (>30% change). The range of BDNF serum values was 15.83–79.77 ng/ml, with a small, positive correlation with age indicating an increase in serum BDNF levels of 0.33% for every year of age (Fig. 2; *p* = 0.031). The values of BDNF content per platelet also indicate a significant, positive correlation with age when pooling the corresponding data (*N* = 475, *p* = 0.03; Fig. 3*A*). Platelet counts also significantly (*p* < 0.001; Fig. 3*B*) correlated with BDNF values with an increase of 14.5% seen with every 100 × 10^9^ increase in platelets. As reported previously (Butkiewicz et al., 2006), there was a significant difference in platelet counts between genders (*p* = 0.0273) with mean platelet counts (geometric mean) of 274.62 × 10^9^ per ml blood for female and 255.62 × 10^9^ for male volunteers. In addition, there is a significant association between BDNF levels and hematocrit (*p* = 0.001; Fig. 4). Mean hematocrit values (geometric mean) were 39.52% for female and 44.04% for male volunteers, reflecting the known gender difference (*p* < 0.001; Vahlquist, 1950). However, no significant gender-dependent differences were observed with regard to the BDNF values in serum.

**Table 1.** Cohort characteristics at Initial (*n* = 259) and 12-month visit (*n* = 226)

	**Visit 1**	**Visit 2**
N	259	226
Age in years [mean (SD)]	44.31 (11.26)	46.11 (10.80)
Sex [males (%)]	81 (31.3)	74 (32.7)
Time between samples in months [mean (SD)]		12.87 (2.21)

**Table 2.** Summary statistics for BDNF values in serum separated by gender

**Variable**	**Gender**	***N***	**Mean (ng/ml)**	**SD (ng/ml)**	***p***
BDNF V1	F	178	32.85	8.57	0.7
	M	81	32.34	7.82	
BDNF V2	F	152	32.98	8.47	0.99
	M	74	32.95	8.19	

**Figure 5.**
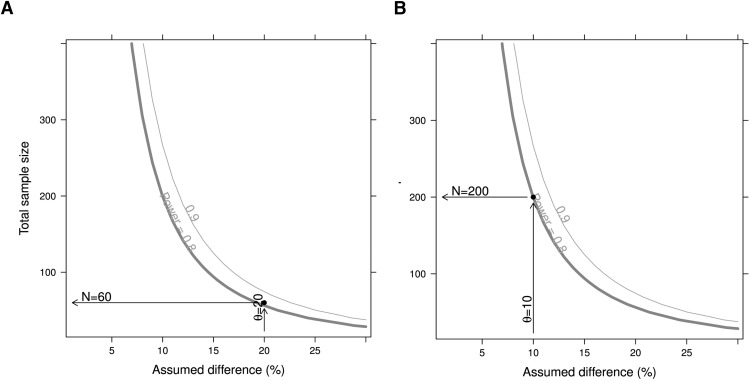
Cohort size estimation as a function of expected difference between two populations. Thick gray lines indicate a power of 80%; fine gray lines indicate a power of 90%. ***A***, Cohort size required for a 20% difference in BDNF values. ***B***, Cohort size required for a 10% difference in BDNF values.

These ELISA results can also be used to estimate the size of the cohorts needed to generate statistically significant results. Assuming equal group sizes, a variability of the measurements of 0.24 (as observed here) and a *t* test of log-normally distributed data with a significance level of 0.05, group sizes of 60 and 200 will be needed to measure group differences of 20% or 10%, respectively, with 80% power (Fig. 5).

**Figure 6.**
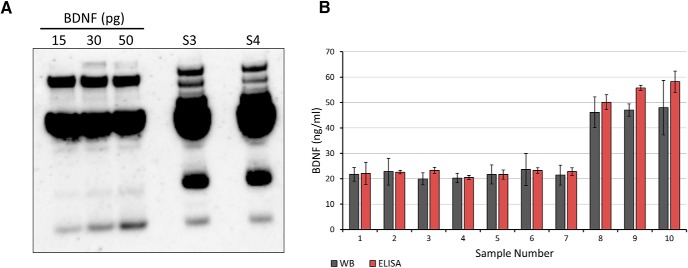
Comparison of mean ELISA values with Western blotting determinations of the same serum samples. ***A***, Western blotting used to quantify BDNF in two different serum samples (1 μl each) against a standard curve generated from three different quantities of recombinant BDNF with added BSA. ***B***, Comparison between mean values obtained by Western blotting versus those obtained by ELISA. Error bars show SD, derived from at least three separate blots (WB) or three replicate wells in the same plate (ELISA).

To validate these ELISA results, a subset of the collected serum samples representing the lower and upper ranges was analyzed by Western blotting (10 samples in total; Fig. 6).

## Discussion

Using the publicly available monoclonal antibodies mAb BDNF-#1 and mAb BDNF-#9 and avidin-coated ELISA plates, mean levels of BDNF in serum were found to be stable over a period of 12 months among the 226 retested volunteers. Within the cohort, single values ranged from a low 15.83 ng/ml to a high 79.77 ng/ml despite a rigorous serum collection protocol. The need to examine large cohorts is further illustrated by the finding that as many as 37 individuals, i.e., ∼16% of the retested volunteers, had values that differed by 20% or more 12 months after the initial samples were collected. Importantly, the mean values reported here could be confirmed by Western blot analysis using a recently developed BDNF monoclonal antibody allowing the detection of BDNF in serum samples without pretreatment. Attempts to deplete samples of major serum proteins, including antibody-mediated adsorption or protein precipitation failed as they led to losses of BDNF of at least 50% when serum levels were determined by ELISA before and after protein depletion. While the mean BDNF serum values reported here fall within the range of a small number of other studies (Shimizu et al., 2003; Lommatzsch et al., 2005; Ziegenhorn et al., 2007), the published average values range from below 1 ng/ml (Onen Sertoz et al., 2008) to over 60 ng/ml (Matrisciano et al., 2009). This variability has been previously explained by issues related to serum collection (Maffioletti et al., 2014; Tsuchimine et al., 2014) and to commercially available antibodies, including antibody precoated plates (Polacchini et al., 2015). In megakaryocytes BDNF is co-stored in α granules with PF4 (Chacón-Fernández et al., 2016) and neither in megakaryocytes nor in pro-platelets did we find any evidence for a cytoplasmic storage of BDNF (Tamura et al., 2011), and it seems that only a fraction of the BDNF content is released during the process of degranulation (Serra-Millas, 2016). This partial release may be a consequence of the dense BDNF packaging in platelets, its association with other components in α granules and/or a consequence of its physico-chemical properties, including its high isoelectric point and hydrophobicity (Barde et al., 1982; Hofer and Barde, 1988). In addition, various commonly used drugs including those known to interfere with the biosynthesis of prostaglandins or of adenosine diphosphate are likely to interfere with the release of BDNF from platelets given their established roles in platelet aggregation (Stoll et al., 2011).

A major reason for the large number of publications reporting on BDNF levels in serum is the intriguing association with numerous conditions including depression (Karege et al., 2002; Sen et al., 2008) and neurodegenerative disorders such as Alzheimer’s disease (Laske et al., 2006; Lee et al., 2009; Forlenza et al., 2010), Parkinson’s disease (Scalzo et al., 2010; Costa et al., 2015; Wang et al., 2016), and Huntington’s disease (Ciammola et al., 2007; Zuccato et al., 2011). Conversely, BDNF levels have also been reported to increase after acute exercise (Rojas Vega et al., 2006; Dinoff et al., 2017). While the reality of an association between the levels of BDNF in serum and any conditions has been questioned (Zuccato et al., 2011), the results of several meta-analyses suggest that these associations may be real (Brunoni et al., 2008; Molendijk et al., 2014; Szuhany et al., 2015). Importantly, post-mortem analyses of BDNF brain content do indicate correlations between brain levels of BDNF and the rate of cognitive decline in diseases such as Alzheimer’s or Parkinson’s (Howells et al., 2000; Buchman et al., 2016) and in depression (Thompson Ray et al., 2011). However, there is still no plausible mechanism satisfactorily explaining how BDNF levels in blood may reflect levels in the brain. The hypothesis that platelets may somehow accumulate BDNF following its diffusion from the brain into the vascular compartment appears unlikely. Beside the lack of BDNF transporter in platelets (Fujimura et al., 2002), intravenous injections of radiolabeled BDNF into rats failed to show any accumulation in the brain, indicating the inability of BDNF to cross the blood-brain barrier (Pardridge et al., 1994). Furthermore, while rats and mice express similar levels of BDNF in the brain, BDNF can be readily detected in platelets and serum of rats, but not of mice. The more plausible source accounting for the presence of BDNF in platelets are the platelet-generating cells, namely megakaryocytes: they contain readily detectable levels of BDNF in rats and humans, but not in mice (Chacón-Fernández et al., 2016). Given the additional finding that neuron-specific *BDNF* transcripts have been detected in megakaryocytes it is conceivable that conditions affecting *BDNF* transcription both in neurons and megakaryocytes may explain correlations between the levels of BDNF in serum and in brain. However, such transcriptional changes are unlikely to explain BDNF increases after acute physical exercise whereby increased release of BDNF from platelets, for example, as a result of their fragmentation or activation, may appear more likely (Posthuma et al., 2015). In general, as is the case for several other growth factors and cytokines contained in platelets, the functional relevance of BDNF in platelets is still unclear though it is intriguing to note that the serum levels of BDNF in all primates tested are comparable to those found in humans (Mori et al., 2003). Accumulation of BDNF in platelets may have conferred a survival advantage in long-lived species, for example by contributing to tissue repair like may be the case for other platelet-derived growth factors and cytokines.

Among the various parameters tested, platelet numbers were found to be the variable most significantly correlated with BDNF serum values, in line with the fact that platelets are the major source of BDNF in serum. However, this correlation was not as strong as may have been anticipated (Fig. 3). Furthermore, while a significant difference in platelet counts was found between genders (*p* = 0.0273) as previously reported (Butkiewicz et al., 2006) no significant differences in the levels of BDNF between genders could be observed, presumably a consequence of the relatively weak correlation between BDNF values and platelet counts. Also, while platelet numbers are known to decrease with age a moderate increase in BDNF serum levels was observed with age (*p* = 0.03; Fig. 3). It thus appears that factors others than platelet numbers also contribute to BDNF levels in serum, including platelet reactivity. For example, it has been previously noted that the release of α granule components such as PF4 increases with age, for reasons that remain unclear (Zahavi et al., 1980).

In conclusion, the results demonstrate that BDNF levels can be reliably measured in human serum samples using publicly available monoclonal antibodies. However, meaningful comparisons require the recruitment of adequately-sized cohorts given individual variations.

## Synthesis

Reviewing Editor: Robert Kalb, Children's Hospital of Philadelphia Decisions are customarily a result of the Reviewing Editor and the peer reviewers coming together and discussing their recommendations until a consensus is reached. When revisions are invited, a fact-based synthesis statement explaining their decision and outlining what is needed to prepare a revision will be listed below.

This is a high quality ms. from an expert in the field. The review points out several places where presentation or discussion of data could be improved. The authors are requested to make this emendations. There is a reasonable request to show new western blot data (i.e., the whole width of the blot as well as the blot from the individual with exceptionally high serum BDNF levels) - please comply

Reviewer's comments:

BDNF is an important regulator of synaptic plasticity in the brain. However, high levels of this neurotrophic factor are also found in human platelets and serum. BDNF serum levels have been determined in many studies to characterize changes in neuropsychiatric disorders such as depression, Alzheimer's disease and others. The relevance of these data is still a matter of debate, but several meta-analyses indicate that reduced BDNF-levels in serum might correlate with disturbed brain function. This paper is a well performed and valid analysis of BDNF serum levels in 259 healthy volunteers determining variability by retesting after 12 months and validation of the ELISA results with Western blot analysis. The results in general are convincing and important for the field. There are some minor issues that could increase the quality of this paper.

1. Abstract: The last sentence "The overall conclusion is that whilst BDNF levels can be reliably measured... comparisons between two populations would only be meaningful if cohorts of sufficient sizes are assembled." The basis for this conclusion is not given in the abstract and the reasons why the authors believe that large cohorts are necessary need to be better introduced in the abstract.

2. Introduction: The authors cite the paper by Koppel et al. 2015 on which start codon is used in neural cells for BDNF translation. The possibility that regulation of translation is different between neurons and megakaryocytes or platelets needs to be mentioned or discussed. The paper by Koppel et al. is not a good reference for translational control of BDNF production in megakaryocytes or platelets.

3. Materials & Methods: The authors have only used 30 min. at room temperature for coagulation of blood, prior to selection of serum. Could longer periods reduce the variation by allowing more BDNF to be released from clotting platelets? This needs to be discussed.

4. The source of recombinant BDNF used as a standard for the Western blot analyses should be given: How did the authors make sure that the levels of BDNF in the three lanes on the left side in the blot shown in Fig. 6a are accurate? This needs to be described and discussed.

5. The authors do not mention how many samples were tested for Western blot analysis. This should be indicated. They mention on p. 5 that blots with significant signal heterogeneity across the width of the BDNF bands were not considered. This is not convincing. All blots should be shown and the whole width demonstrated and discussed in this paper to avoid selection bias. It would also be particularly interesting to see a blot from a patient sample with exceptionally high levels of BDNF in serum, in order to determine whether these levels can be validated or whether so far unknown factors could influence ELISA results and lead to erroneously high BDNF levels.

6. Results section: The variability should not be given as percent levels for the coefficient of variability. The whole range should be mentioned. This is important to draw conclusions on the number of samples and replications which are necessary to obtain reliable results from ELISA assays.

7. Discussion: On p. 8 the authors write that "BDNF is stored in platelets in alpha granules where it is co-stored with PF4 (Charcon-Fernandez et al. 2016)". However, this paper focused on megakaryocytes and showed that BDNF is present in alpha granules in this cell type, but this paper did not include evidence for platelets. This needs to be corrected because it is far from clear whether BDNF is exclusively located in alpha granules within platelets. The study by Serra-Millas 2016 discusses in detail that only about 30% of total BDNF levels within human platelets is stored in alpha granules and a relatively high proportion of BDNF is found in the cytoplasm of platelets. Despite the fact that the underlying evidence is not fully convincing, this raises important questions that require further detailed analysis. Therefore, this needs to be discussed in more detail.

## Author Response

Rebuttal letter

We are grateful for the helpful, high-quality comments regarding our submission and address them all in the following paragraphs:

Visual Abstract

We suggest using a simplified version of Fig. 1 as it summarises the key findings, in particular the large number of volunteers tested, the relative stability of the values over time and the range of BDNF levels in serum of this large cohort. As it is self-explanatory and easy to grasp we are thankful for the suggestion as our manuscript may then receive more attention.

Detailed comments:

1. Abstract: The last sentence “The overall conclusion is that whilst BDNF levels can be reliably measured... comparisons between two populations would only be meaningful if cohorts of sufficient sizes are assembled.” The basis for this conclusion is not given in the abstract and the reasons why the authors believe that large cohorts are necessary need to be better introduced in the abstract.

This conclusion is now explicitly supported in the Abstract. We added the following sentence ''different (N=226, 32.97 Â± 8.36 ng/ml SD, p=0.19). Power analysis of these values indicates that relatively large cohorts are necessary to identify significant differences, requiring a group size of 60 to detect a 20 % change. The levels determined''

2. We recognise that referencing the work by Koppel et al. in this particular context may not be useful given that our study does not address the translational control of BDNF. This reference has now been deleted.

3. Materials & Methods: The authors have only used 30 min. at room temperature for coagulation of blood, prior to selection of serum. Could longer periods reduce the variation by allowing more BDNF to be released from clotting platelets? This needs to be discussed.

This is an important point already highlighted the importance of this (see Maffioletti et al 2014 in the list of references). In the current study, we used collection tubes with clotting activator, which have an optimal incubation time of 30 minutes according to manufacturer's instructions. A sentence has been added into the Methods section to make it clear this was the case ('Serum Samples', line 7): ''S-Monovette 7.5 ml Z tube (with clotting activator; Sarstedt), left to coagulate for 30 min at room temperature''

4. The source of BDNF is now indicated (E. Coli BDNF provided by Regeneron/Amgen partner). The same stock solution is used for BDNF quantification by ELISA and Western blot using dilutions in protein-containing solutions as indicated. With regard to the accuracy of the determination the linearity of the signal has been checked in every single experiment and the results used for quantitative determinations only when the r2 value was >0.98. Note that the same criteria were applied to the ELISA determinations (see also #5).

5. The authors do not mention how many samples were tested for Western blot analysis. This should be indicated. They mention on p. 5 that blots with significant signal heterogeneity across the width of the BDNF bands were not considered. This is not convincing. All blots should be shown and the whole width demonstrated and discussed in this paper to avoid selection bias. It would also be particularly interesting to see a blot from a patient sample with exceptionally high levels of BDNF in serum, in order to determine whether these levels can be validated or whether so far unknown factors could influence ELISA results and lead to erroneously high BDNF levels.

The sentence referring to 'signal heterogeneity' referred to obvious transfer problems (e.g. air bubbles) and as it seems to have been ambiguous it has now been removed. As Western blot validation of the ELISA results is one of the key novel points of the paper we have included additional data in Figure 6 showing the ELISA and WB values obtained from samples with much higher BDNF levels (50 ng/ml range, compared with those illustrated previously in the 20 ng/ml range).

We have now added in the number of samples tested by western blot ('Results', line 37): 'To validate these ELISA results, a subset of the collected serum samples representing the lower and upper ranges was analysed by Western blot (10 samples in total; Figure 6).'. However, we were puzzled by the comment related to the illustration of the Western blot as the illustration provided showed everything that there is to see. The misunderstanding may be explained by the fact that samples were only loaded every other lane (because of the high protein levels in serum samples). Ten-well gels were used for these western blots, 5 containing standards or samples and 5 'empty' wells (loaded with 1 x LDS), of which two can be seen in Figure 6.

6. Results section: The variability should not be given as percent levels for the coefficient of variability. The whole range should be mentioned. This is important to draw conclusions on the number of samples and replications which are necessary to obtain reliable results from ELISA assays.

The inter-assay variability range has now been added into the text ('Results', line 4): 'This assay has an inter-assay coefficient of variability of 8.8 % as calculated with a single serum sample measured on 18 different plates (24.11 Â± 2.12 ng/ml (SD); range 20.63 - 28.66 ng/ml). The intra-assay coefficient of variability was calculated as 9.7 % based on 253 samples tested in triplicate.'

For the intra-assay coefficient of variability we feel that it would be less useful to include these individual values in the manuscript as this would mean adding triplicate values of over 500 samples.

7. Regarding the localisation of BDNF, our previous work did indeed deal with megakaryocytes, not platelets but please note that we also illustrated the localisation of BDNF in so-called pro-platelets. The signal is not different from what we document in megakaryocytes to be localised in α-granules using PF4 as marker. Our experiment failed to

reveal a cytoplasmic staining in pro-platelets. Still, as there is a possibility that the localisation of BDNF in platelets may not be the same as in megakaryocytes we reformulated this paragraph to eliminate possible ambiguities. We understand the caution expressed by the reviewer to be based on the review by Serras-Millas quoting work by Tamura and colleagues (Thrombosis Research 128, 55-61, 2011). However we question the specificity of the antibody used by Tamura and colleagues as their illustrations indicate two different molecular weights for BDNF as a function of its presence in or outside α-granules. The BDNF monoclonal antibody we used failed to reveal different molecular weights for BDNF in platelet lysates as indicated in the illustration we provided in Chacon-Fernandez et al., 2016). Likewise the immuno EM pictures provided by Tamura have been obtained with polyclonal antibodies of unclear specificity - we indicated in our previous work with brain sections that none of the commercially available antibodies we tested are suitable for immunostaining (when appropriately controlled with brain tissue lacking BDNF). In any event we changed the discussion related to the release of BDNF from platelets (see #3 above) as ultimately it is still unclear why only a fraction of BDNF stored in platelets seems to be released by the process of platelet degranulation.
